# Milia En Plaque After Bullous Pemphigoid: An Unusual Sequela

**DOI:** 10.7759/cureus.73992

**Published:** 2024-11-19

**Authors:** Helen Z Chen, Mary A Elhawi, Michael A Selby, Michelle Tarbox

**Affiliations:** 1 Dermatology, Texas Tech University Health Sciences Center, Lubbock, USA; 2 School of Medicine, Texas Tech University Health Sciences Center, Lubbock, USA

**Keywords:** autoimmune blistering diseases, bullous pemphigoid, dermoscopy, milia, milia en plaque

## Abstract

We present the case of a 36-year-old paraplegic woman with a history of spinal cord injury who developed a generalized blistering rash, later diagnosed as bullous pemphigoid (BP). During her hospitalization, she was treated with prednisone and rituximab infusions, transitioning to maintenance therapy with topical steroids, doxycycline, and nicotinamide. A year later, she presented with concerns about a BP flare on her feet. Examination revealed 1-2 mm white, dome-shaped papules coalescing into erythematous plaques on the plantar surfaces, leading to a diagnosis of milia en plaque (MEP). Treatment with tretinoin 0.05% cream resulted in near-complete resolution within six months. MEP is a rare but benign condition characterized by keratinous cysts on erythematous plaques, often linked to immunobullous disorders such as epidermolysis bullosa acquisita or mucous membrane pemphigoid, and only rarely associated with BP. Distinguishing MEP from active BP is critical, as the latter requires immunosuppressive therapy, while MEP can be managed conservatively. This case underscores MEP as a sequela of BP, highlighting the importance of accurate diagnosis and appropriate management.

## Introduction

Multiple milia, also known as milia en plaque (MEP), is a rare benign dermatosis characterized by multiple milia cysts within an inflammatory plaque [1]. MEP most commonly affects the face, particularly the cheeks and eyelids, and is classified into primary and secondary forms. Primary milia arise spontaneously, whereas secondary milia result from trauma or underlying skin conditions such as burns, dermabrasion, infectious causes like herpes zoster, and immunobullous diseases [2].

It is hypothesized that milia formation secondary to blisters occurs through the regeneration of sweat glands or hair follicles [3]. In immunobullous diseases, milia typically develop during the recovery phase of conditions such as mucous membrane pemphigoid (MMP) and epidermolysis bullosa acquisita (EBA) [4]. Although rare, multiple milia have also been observed following bullous pemphigoid (BP) [5]. Notably, this phenomenon has been uniquely associated with the HLA-DQ6 antigen, suggesting an immunological predisposition to milia formation during BP recovery [6].

The BP 180 antigen indirectly interacts with type VII collagen via laminin-332 and type IV collagen. Abnormal interactions between hemidesmosomal proteins and extracellular matrix components beneath the hemidesmosomes may contribute to milia formation [3].

This case report seeks to highlight MEP as a distinct clinical entity with specific treatment options and its potential occurrence as a sequela of BP. Proper recognition and differentiation from active BP flares are crucial for appropriate management.

## Case presentation

**Figure 1:**
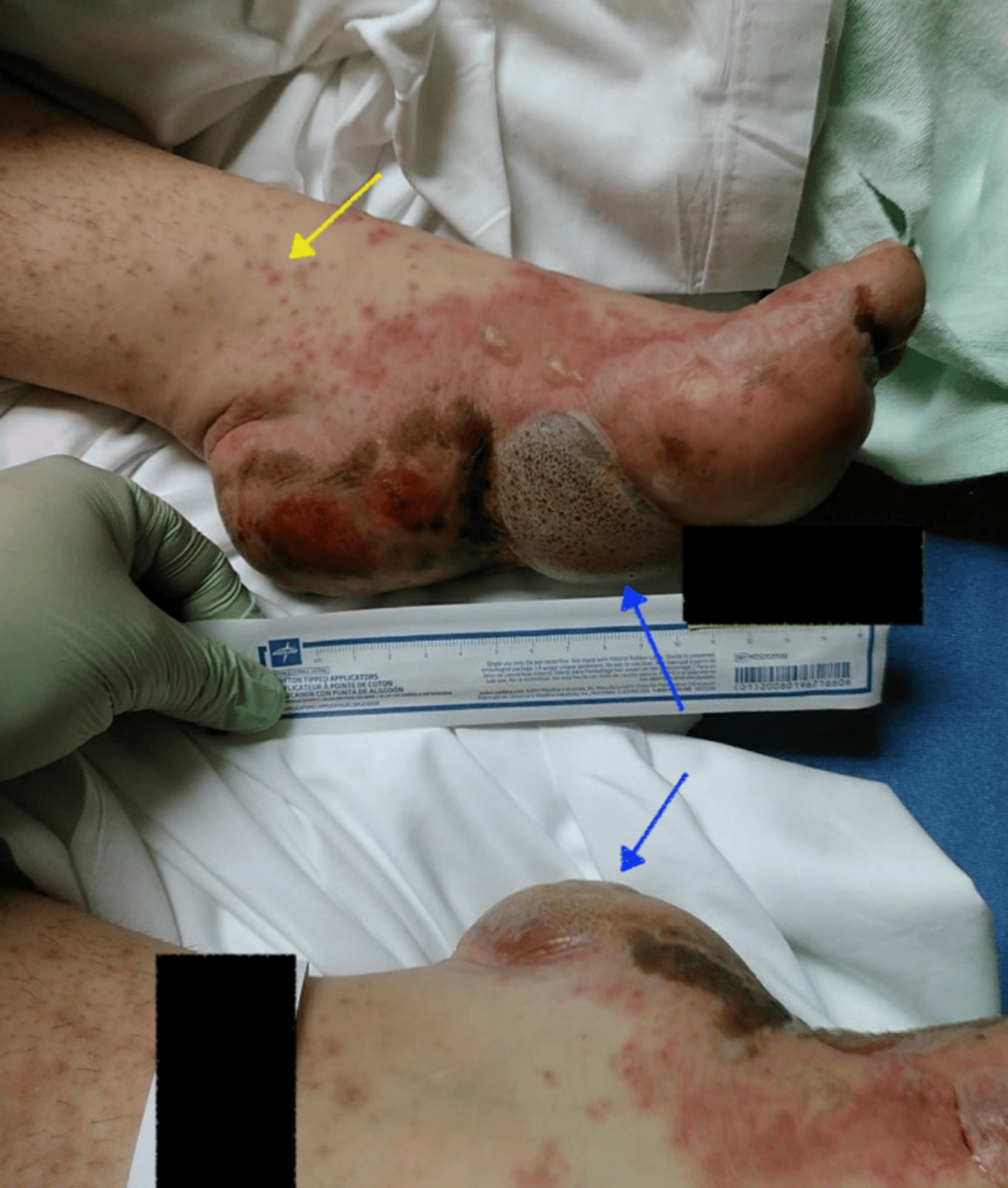
Erythematous pinpoint papules (yellow arrow) are visible along the ankles and medial feet, accompanied by clear to erythematous and purpuric blisters and bullae (blue arrows) on the plantar surfaces

**Figure 2:**
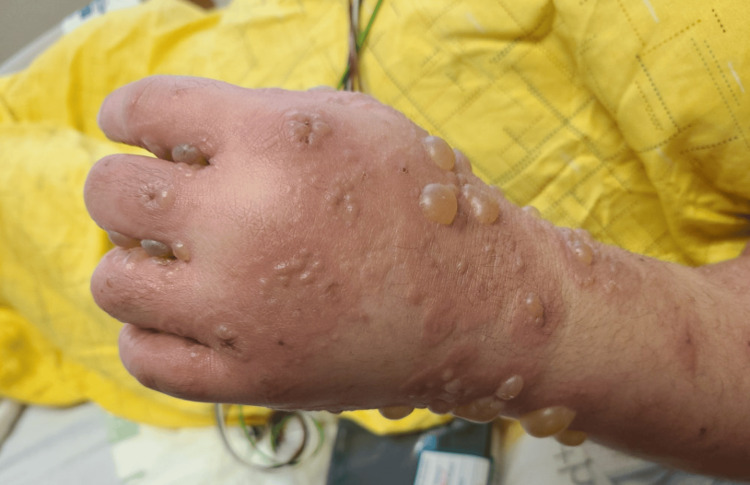
Multiple clear to purpuric vesicles and bullae are observed on the left hand and forearm, overlying an erythematous urticarial base

**Figure 3:**
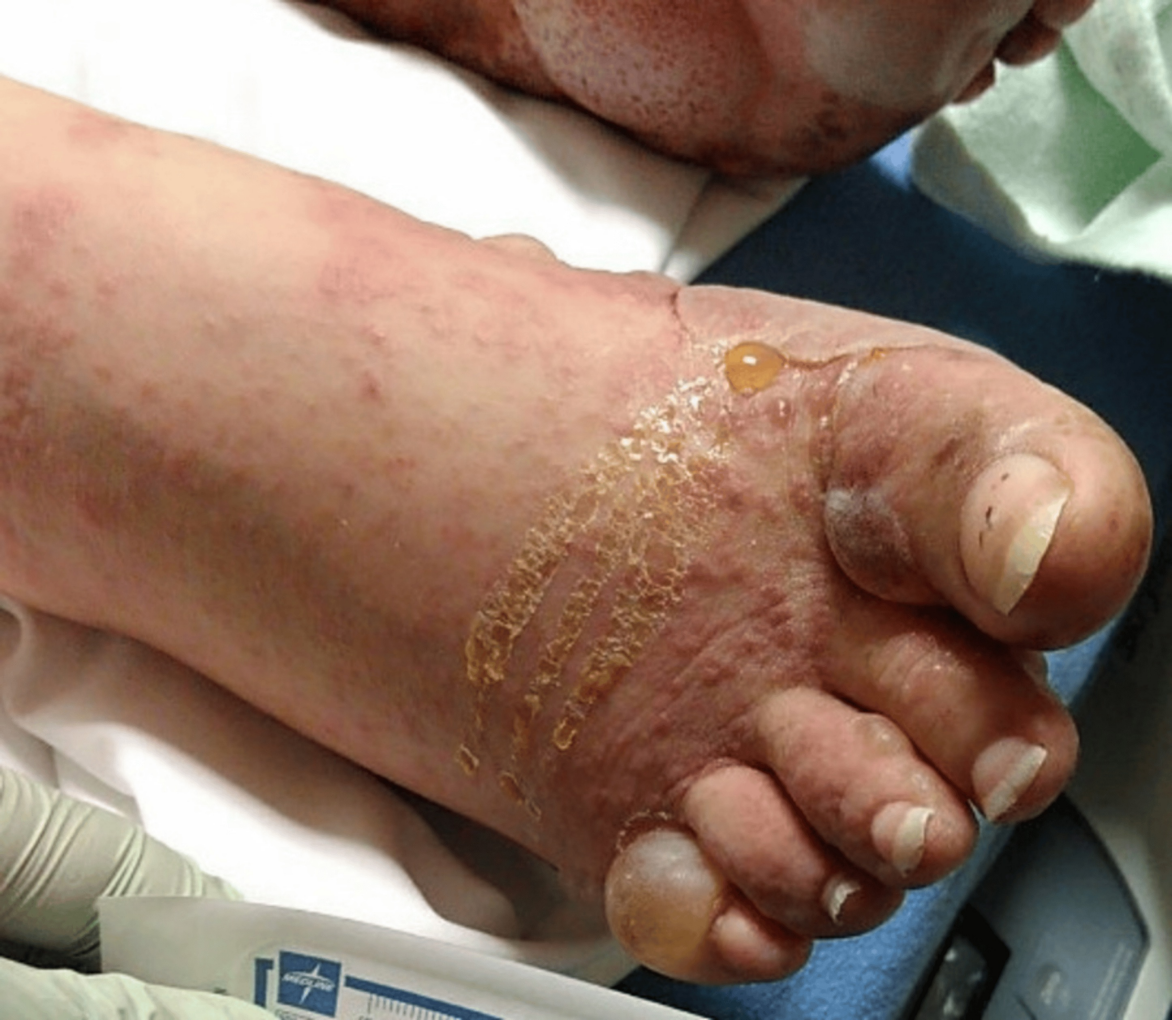
Erythematous papules with honey-colored crusting, accompanied by clear blisters and bullae, are observed on the dorsal surfaces of the right foot

**Figure 4:**
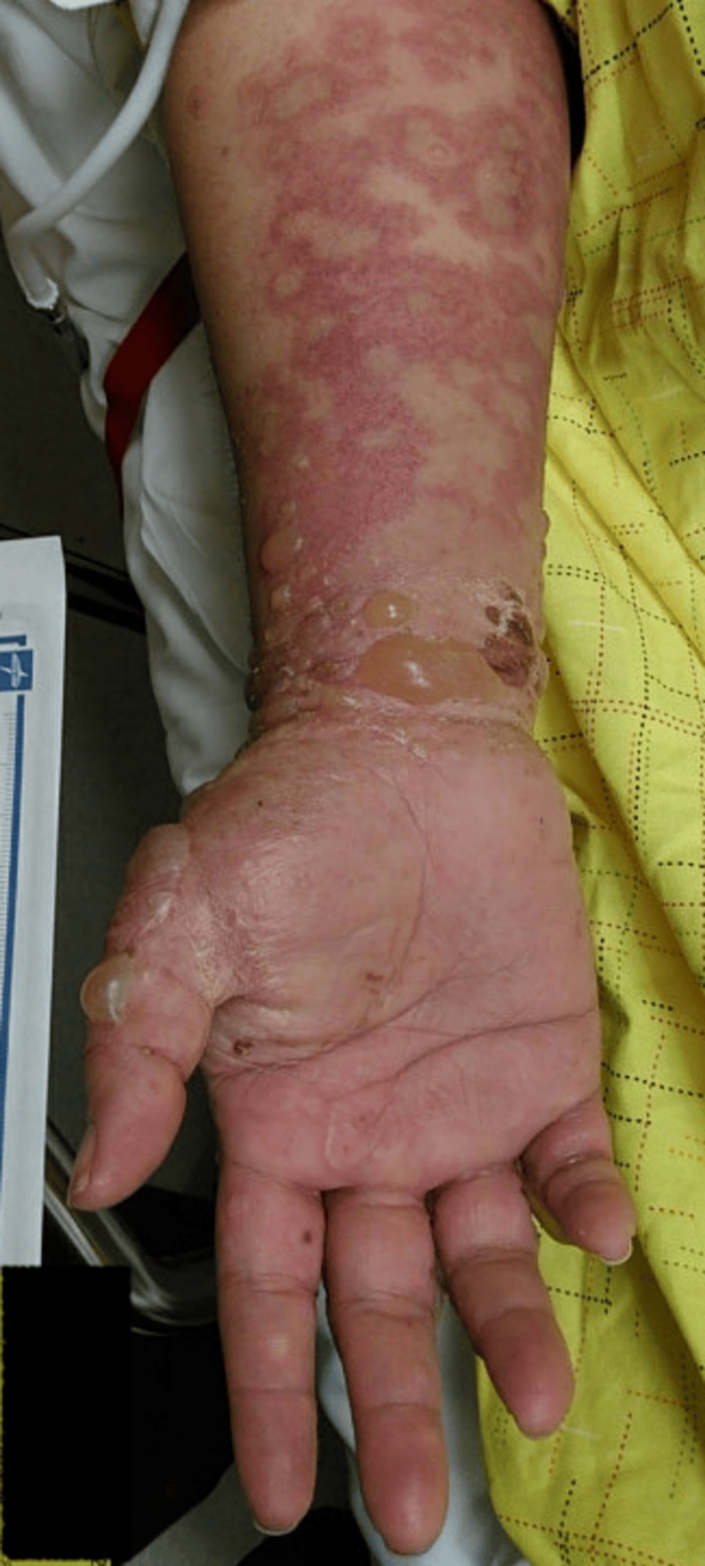
Erythematous urticarial wheals are observed on the forearm, accompanied by blisters and bullae on the wrist and fingers

We present the case of a 36-year-old female with a history of paraplegia secondary to a spinal cord injury from a motor vehicle accident. During her initial hospitalization, she presented with a blistering rash that began as small bumps on the arms and thighs, which subsequently spread to the hands, feet, trunk, and face, eventually developing into blisters and bullae, as shown in Figure 1, Figure 2, Figure 3, and Figure 4.

A biopsy during her inpatient hospitalization revealed a subepidermal blister with numerous eosinophils and neutrophils, along with a superficial perivascular lymphocytic and eosinophilic infiltrate in the dermis. Direct immunofluorescence demonstrated linear deposition of IgG and C3 at the dermal-epidermal junction, and her ELISA BP-180 antibodies were elevated at 193, consistent with the diagnosis of BP. The patient’s treatment plan initially included prednisone at 0.5 mg/kg, but due to worsening skin involvement, she was ultimately treated with two 1 g infusions of rituximab, administered 15 days apart. Additionally, she was prescribed nicotinamide 1500 mg daily, doxycycline 100 mg twice daily, and clobetasol ointment 0.05% as needed for affected areas.

**Figure 5:**
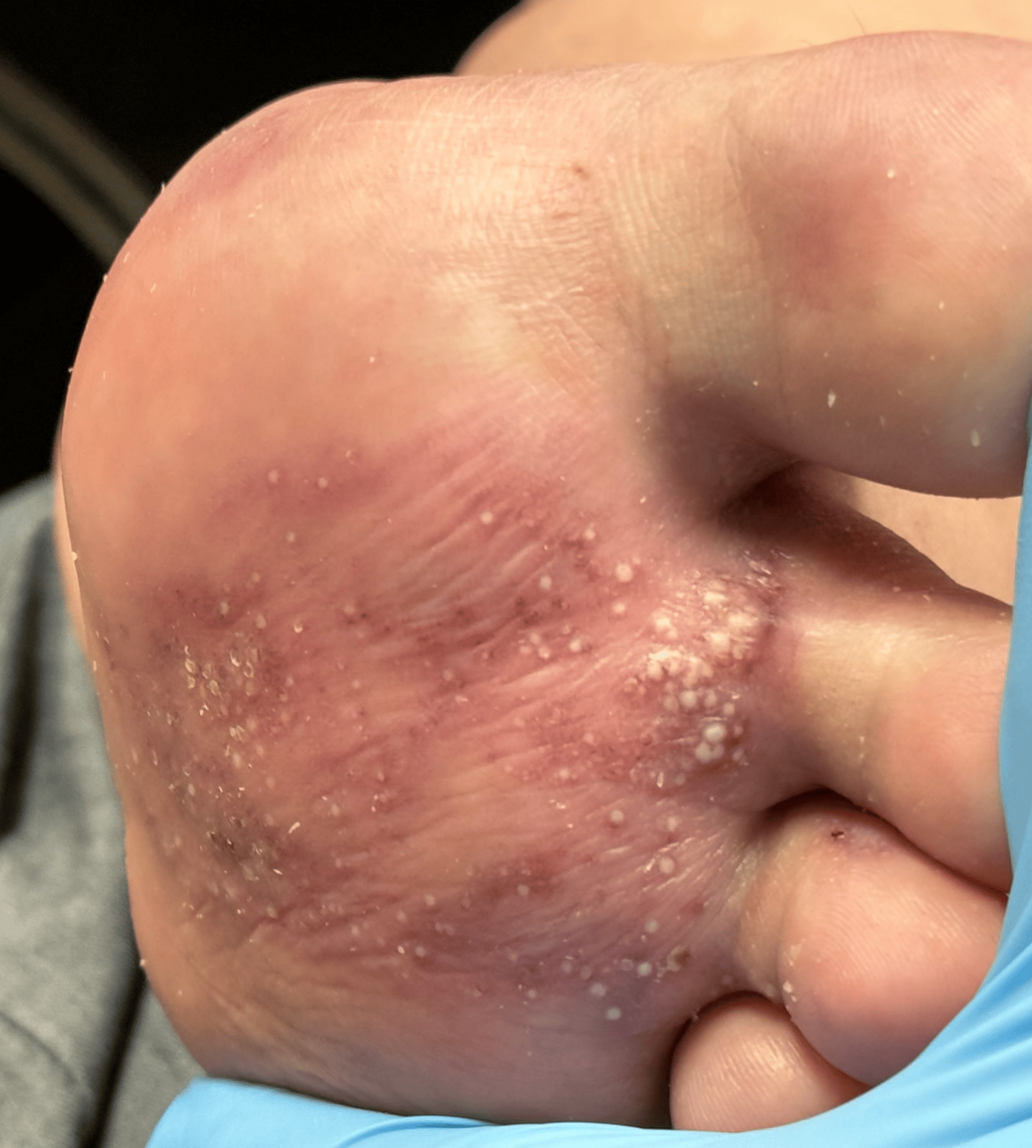
1-2 mm white, pearly papules coalescing into an erythematous plaque are observed on the plantar surfaces of the feet

**Figure 6:**
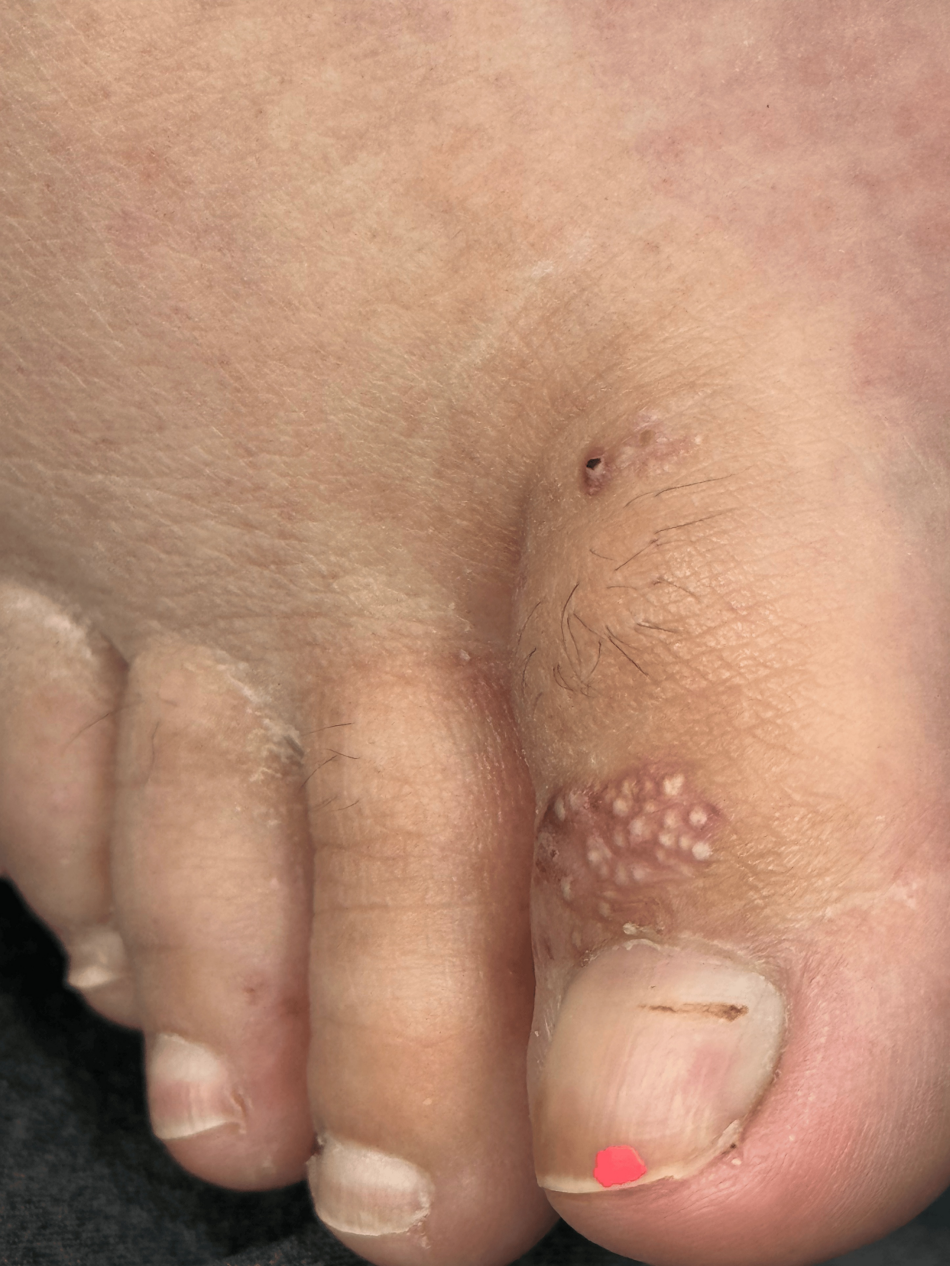
White papules on an erythematous base are seen on the dorsal aspect of the first digit, near the distal interphalangeal joint

**Figure 7:**
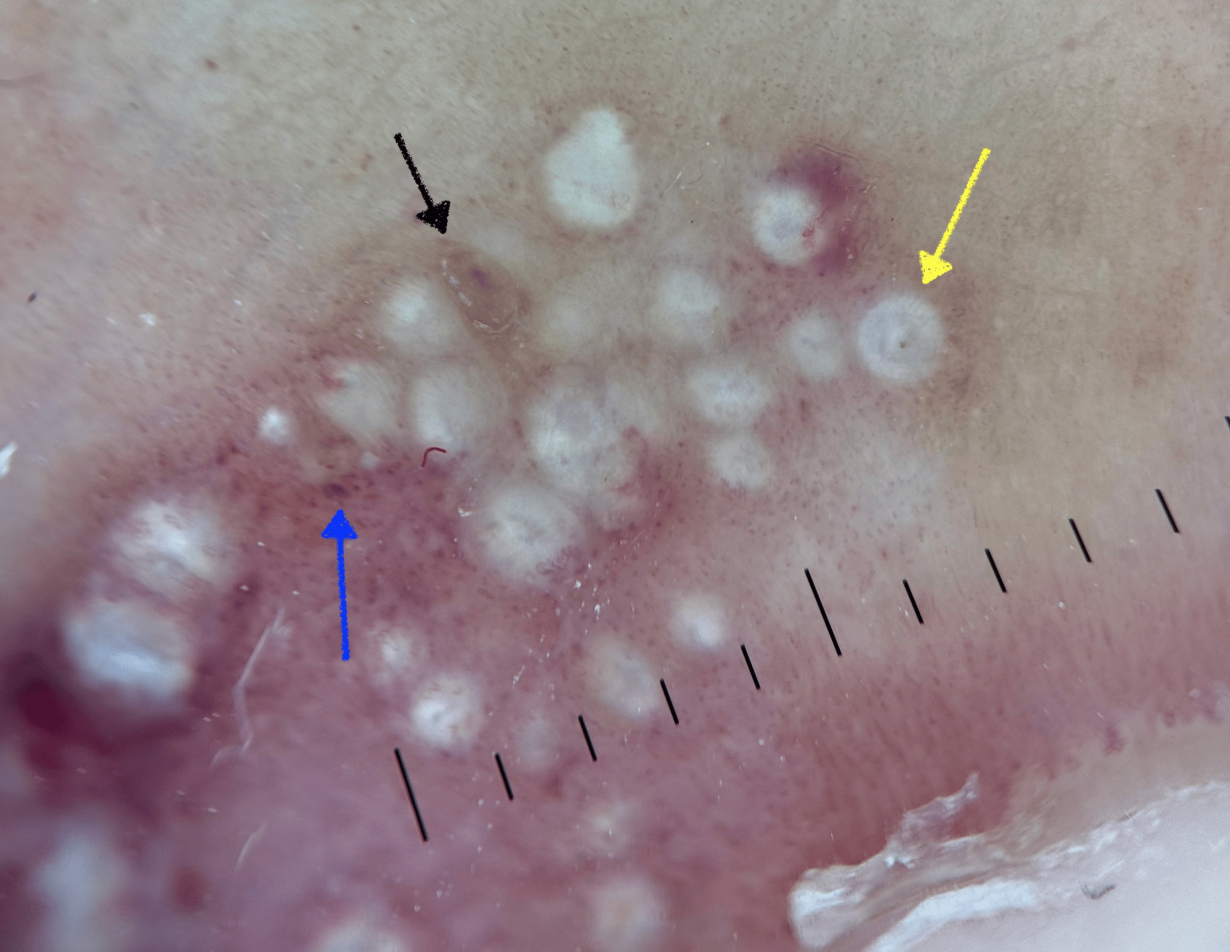
Dermoscopic image of MEP showing numerous small white papules (yellow arrow), telangiectatic blood vessels (blue arrow), and multiple brown globules (black arrow) MEP, milia en plaque

A year later, she presented to our clinic with concerns of a BP flare on her feet. On physical examination, 1-2 mm white, dome-shaped, pearly papules were observed, coalescing into an erythematous plaque along the bilateral plantar surfaces of the feet and between her toes, with the left foot more severely affected than the right foot, as shown in Figure 5 and Figure 6. On dermoscopy, numerous small cysts of varying sizes, along with scattered brown irregular speckles and telangiectatic blood vessels, were observed, as shown in Figure 7. These findings are consistent with previous dermoscopic findings of MEP [7].

**Figure 8:**
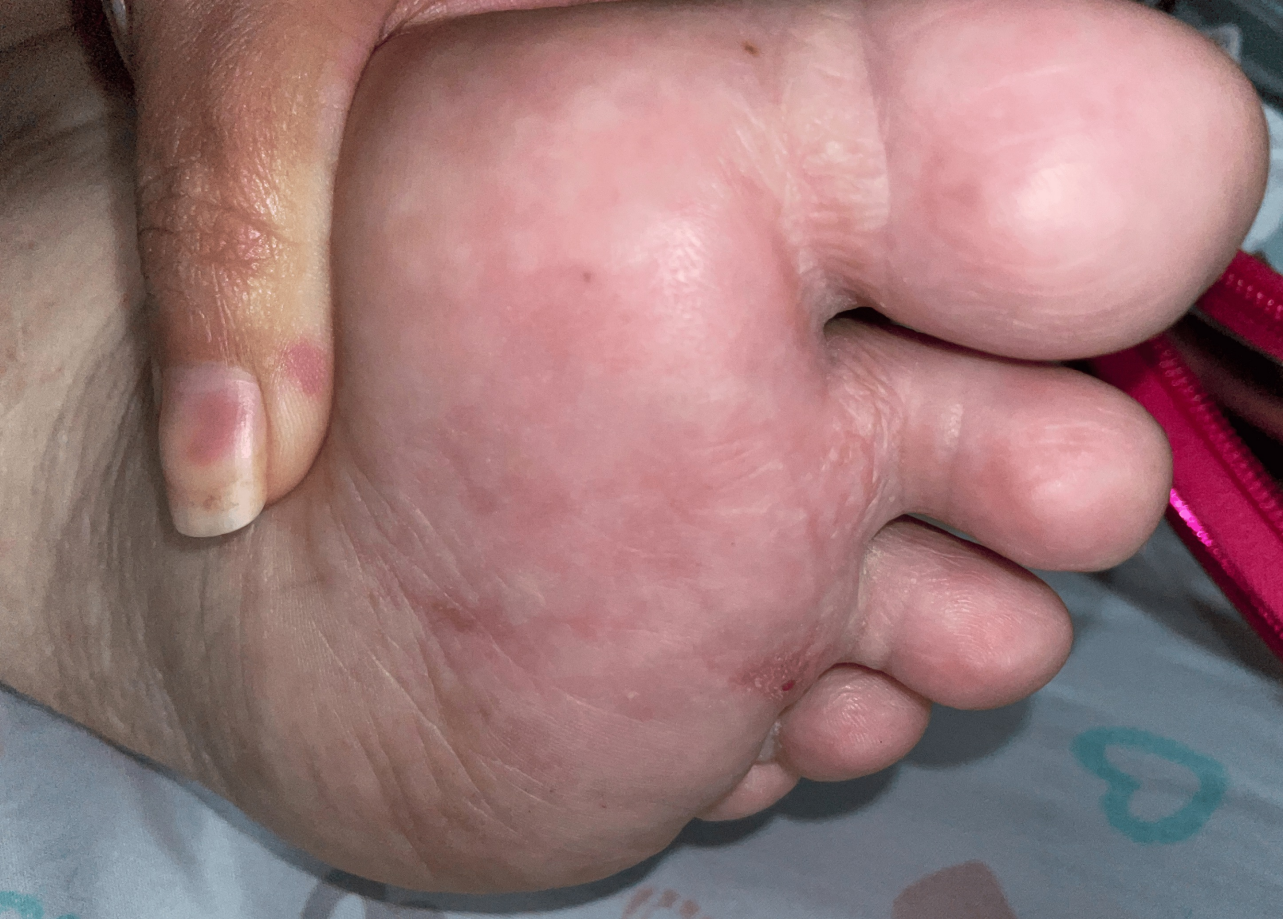
Post-inflammatory hypopigmented macules are observed on the plantar surfaces of the feet following the resolution of MEP MEP, milia en plaque

A clinical diagnosis of MEP was made, and the patient was closely followed to ensure no exacerbation of her underlying BP. She was started on tretinoin 0.05% cream nightly, and at the six-month follow-up, only the first digit of her left foot showed involvement, with five 1-2 mm white, dome-shaped pearly papules, while the other areas of her bilateral feet had cleared completely (Figure 8).

## Discussion

MEP is a rare benign dermatosis that clinically presents as an erythematous plaque with numerous keratinous cysts [7]. While milia secondary to blisters are more commonly associated with EBA and MMP, they are rarely observed in BP [4]. This underscores the importance of recognizing MEP as a distinct entity that can occur following a BP flare and should be differentiated from an active BP flare. Milia cysts are superficial, dome-shaped, and pearly white, with a diameter ranging from 1-4 mm [8]. Histologically, multiple infundibular cysts lined by stratified squamous epithelium are found throughout the dermis [1]. Dermoscopically, MEP presents with multiple white-yellow papules, spotty brown pigmentation, and telangiectasias over an erythematous base [7]. In contrast, dermoscopy of BP reveals greater variability, including yellow-pink translucent areas with a distorted pigment network, as well as prominent follicular and eccrine openings surrounded by pigmentation [9].

It is crucial to differentiate between an active BP flare and MEP, as treatment for BP typically involves immunosuppressive medications, while MEP often requires only reassurance. For patients seeking treatment for MEP, several options are available, including topical tretinoin, oral etretinate, photodynamic therapy with methyl aminolevulinate hydrochloride, erbium-YAG laser, fractional CO2 laser therapies, radiofrequency, electrodesiccation, and microwave thermotherapy [1]. Topical retinoids have been shown to result in significant clearance in several case reports, although no standardized therapy has been established [1]. In our case, the patient was advised to apply 0.05% tretinoin nightly, which led to near-complete clearance of her MEP, while maintaining nicotinamide and doxycycline therapy for BP.

MEP is a benign and often asymptomatic condition with an excellent prognosis, and treatment is generally pursued for aesthetic reasons. In contrast, the prognosis for BP depends on factors such as age, Karnofsky score (a measure of physical activity), and disease severity at presentation. BP carries a much more serious prognosis, with a 1-year mortality rate ranging from 25% to 40% [10]. A small descriptive study in Brazil found that 87.5% of patients in the BP-milia cohort had elevated total IgE levels, an important finding since increased IgE levels have been linked to greater BP severity [11]. For patients with significant blistering and bullae involvement in BP, clinicians should counsel them about the potential for milia formation following the healing of blistered lesions.

This case highlights the rare association between MEP and BP. By recognizing MEP as a distinct medical sequela rather than an active flare of BP, clinicians can avoid unnecessary escalation of immunosuppressive therapy and provide reassurance regarding a benign condition that may occur after a severe BP flare. This case aims to raise awareness of this unique entity, supported by dermoscopic images and available treatment options.

## Conclusions

We report a case of MEP following BP, a benign sequela that is important to distinguish from an active flare of BP. Although milia formation has been more commonly observed after EBA and MMP, it is rarely associated with BP. Given the limited number of cases reported in the literature, our case highlights the value of clinical photography and dermoscopy in accurately diagnosing MEP. By recognizing the patient’s condition as MEP rather than an active BP flare, we were able to avoid unnecessary escalation of immunosuppressive therapy. After six months of treatment with topical tretinoin 0.05% cream, the patient’s lesion showed near-complete resolution, with ongoing follow-up to manage her primary condition, BP.
